# Make Fitness Fun: Could Novelty Be the Key Determinant for Physical Activity Adherence?

**DOI:** 10.3389/fpsyg.2020.577522

**Published:** 2020-10-15

**Authors:** Nemanja Lakicevic, Ambra Gentile, Samira Mehrabi, Samuel Cassar, Kate Parker, Roberto Roklicer, Antonino Bianco, Patrik Drid

**Affiliations:** ^1^Program in Health Promotion and Cognitive Sciences, University of Palermo, Palermo, Italy; ^2^Department of Psychology, Educational Science and Human Movement, University of Palermo, Palermo, Italy; ^3^Department of Kinesiology, Faculty of Applied Health Science, University of Waterloo, Waterloo, ON, Canada; ^4^School of Exercise and Nutrition Sciences, Institute for Physical Activity and Nutrition, Deakin University, Geelong, VIC, Australia; ^5^Faculty of Sport and Physical Education, University of Novi Sad, Novi Sad, Serbia

**Keywords:** exercise novelty, flow state, motivation, sport participation, exercise prescription, health benefits

## Introduction

### The Determinants of Individuals' Physical Activity Engagement Over Time

The benefits of physical activity (PA) are well known and are extensively delineated in the scientific literature. Regular participation in PA, or exercise as its subset (structured, preplanned form of PA), is positively associated with numerous physical and psychological health benefits across all population subgroups (i.e., different age groups, gender, ethnicity, and socioeconomic status) (Paterson and Warburton, 2010; Warburton and Bredin, 2017). The current PA guidelines for adults, proposed by the American College for Sports Medicine and American Heart Association (ACSM/AHA), recommend the accumulation of at least 150 min of moderate intensity aerobic PA per week (Nelson et al., 2007). Additionally, it is recommended that adults should engage in muscle-strengthening activities at least two times per week (Garber et al., 2011). Despite this, sedentary behavior remains a major challenge, and insufficient PA rates have remained stable at the global level between 2001 and 2016 (Guthold et al., 2018). Physical inactivity is the fourth leading cause of death worldwide and its annual health-care costs have been estimated at 53.8 billion US dollars in 2012 internationally (Ding et al., 2016). However, it has recently been shown that with sufficient PA, as many as 3.9 million premature deaths could be averted annually (Strain et al., 2020), and thus more needs to be done to improve engagement and maintenance of PA throughout the lifespan.

The correlates and determinants of PA participation and maintenance are complex and diverse, and differ among various population subgroups (Thiel et al., 2018; Parker et al., 2019). This can be best described using an ecological framework which posits that there are multiple levels of influence on an individual's PA behavior including: individual (i.e., motivation, competence, preference, and self-efficacy), environmental (including the social, natural, and built environment) and policy level factors (Bauman et al., 2012). It follows that these factors are the key pillars of PA adherence, which is defined as a habitual participation in PA on a voluntary basis (Robison and Rogers, 1994).

A lack of novelty regarding an individuals' exercise routine may also be a key factor contributing to low PA participation in the general population (Dalle Grave et al., 2011). Even among active individuals, continued engagement in the same exercise over time can halt progressive overload and potentially contribute to reversibility (retraction to baseline condition) (American College of Sports et al., 2018), and can ultimately reduce motivation to participate in PA out of boredom. This seemingly vicious cycle can possibly be tackled by introducing novel exercise regimens on a regular basis.

Generally, PA is distinguished in terms of the major metabolic pathway involved in energy contribution, predominantly aerobic or anaerobic. Aerobic and anaerobic training have shown to have positive effects on cardiovascular health (Patel et al., 2017), both separately and when combined. Indeed, the majority of PA modes include a mixture of both, thereby allowing participants to obtain benefits of both types of PA. Additionally, the introduction of new exercises in the PA routine will lead to novel skill acquisition and mastery necessary for that particular PA (Green and Bavelier, 2009). Notably, engaging in new gestures can further develop an individual's skillset, enabling an expansion of the spectrum of PA options one can participate in. Furthermore, exposing the body to a variety of novel activities leads to improvements in body composition and fitness among children (Liu et al., 2020) and adults (Wilhelm and Pinto, 2019). To this regards, youth who experience multiple sports, as a subset of PA, increase their scope for varied PA in adulthood and these experiences may foster an intrinsic motivation for lifelong participation (Mostafavifar et al., 2013). Meanwhile, as the “feel good” effect of exercise is known to differ according to intensity among individuals (Ekkekakis and Brand, 2019), varied PA participation may be an important pre-requisite for finding preferred and tolerable PA options which in turn may lead to longer term adherence. These findings underscore the importance of understanding not only the physiological effects of PA, but also the need to account for how varied PA is experienced in relation to pleasure and enjoyment. Therefore, based on the aforementioned, the aim of this work is to highlight novelty as potentially a key determinant for PA adherence.

### The Psychological Features of Exercise Novelty

Few studies about the role of novelty in PA adherence exist. It is well known that intrinsic motivation predicts the PA adherence through interest (the desire to engage and expand skills), and enjoyment (the desire to have fun and pursue interest) (Richard et al., 1997; Teixeira et al., 2012; Gardner and Lally, 2013).

According to Berlyne (1970), novel stimuli may promote interest and enjoyment when they are simple, while familiar stimuli arouse interest when they contain variations that create complexity in movement variations (Sylvester et al., 2018). Therefore, performing new and challenging exercises could increase enjoyment and interest while enhancing acquisition of new skills every time a new exercise is introduced. Moreover, it has been recently hypothesized that the need for novelty, that is the need to experience something that stands out of the routine (González-Cutre et al., 2019), could be one of the Basic Psychological Needs (Bagheri and Milyavskaya, 2020; Fernández-Espínola et al., 2020). In this sense, creating programs satisfying the need for novelty, and stimulating interest and enjoyment at the same time, could also promote individuals' participation over time, through the introduction of new exercises in the PA routine, or the variation of familiar exercises (Sylvester et al., 2018). For example, fitness regimes such as CrossFit, whereby participants are exposed to constantly varied functional movements, are shown to elicit higher levels of satisfaction and motivation for engagement (Claudino et al., 2018). Nevertheless, since the repeated exposure to the same stimulus could lead to a decrease in interest and enjoyment, the introduction of novel exercise should be alternated with the familiar complex exercise (Sylvester et al., 2018). Finally, enhancing intrinsic motivation through interest and enjoyment is expected to increase adherence to the PA program (Richard et al., 1997), leading to better health outcomes (Robison and Rogers, 1994).

One interesting outcome of performing new exercises, which also reinforces an individual's level of engagement, is the flow experience. Flow experience is described as a sense of concentration and absorption, with suppression of irrelevant feelings and thoughts, resulting in a deep engagement in that activity (Csikszentmihalyi and Csikszentmihalyi, 1992; Csikszentmihalyi, 2020). This state is also related to future involvement in the same situation, and can lead to increased autonomous motivation, and a reduction of boredom. In a recent study by Swann et al. (2019), the authors explored the predictors of flow state in athletes, and found that this phenomenon is more likely to occur in the context of novelty, exploration, and flexible outcomes. Therefore, flow state is induced when exercises are new, or are presented with some variations, and when low pressure or importance is placed on the outcome. In summary, the exercise features that may lead to sustained high PA engagement include novelty, challenging variation of familiar exercises, and flexible outcomes.

## Discussion

Engagement in long-term PA has many psychological, psychosocial and physical health benefits (Both et al., 2010; Warburton and Bredin, 2017). However, individuals' PA participation often decreases over time due to various environmental or personal factors (Calvo et al., 2010). One critical determinant ensuring long-term engagement in PA could be the reinforcement of intrinsic motivation, through interest and enjoyment. Physical activity regimens comprised of new exercises or varying familiar exercises could rise participants' interest and enjoyment and enhance their adherence to PA, ultimately leading to better health.

For example, interactive video-game systems that combine gaming and PA (also known as exergames), and exercise programs delivered *via* digital platforms (e.g., smartphone apps) also are plausible alternative strategies to encourage PA participation, and improve quality of life (Sween et al., 2014; Romeo et al., 2019). Exergaming through novel mediums such as virtual reality (VR), and digital behavior change apps provide a multisensory and immersive environment that is customizable to the user's functional abilities and personal preferences, and thus may increase motivation for regular PA participation or enhanced adherence (Albergoni et al., 2019; Ng et al., 2019; Stockwell et al., 2019; Bonato et al., 2020). For individuals reluctant to participate in PA, the interactive environment of exergames can create a distraction from negative thoughts about PA and encourage the individual to participate in a non-traditional environment (Molina et al., 2014; Street et al., 2017). Further, apps that include social networking and media sites offer the additional benefits of social interaction and support shown to increase PA enjoyment and adherence (Ferrer and Ellis, 2017; Petersen et al., 2019). These platforms may be particularly useful in the current era of the COVID-19 pandemic where governments around the world have imposed quarantine measures to reduce the risk of COVID-19 infection which inevitably leads to lower PA levels in the overall population (Lakicevic et al., 2020), particularly among those who were already inactive (Hall et al., 2020). Even though the long-term of effects of COVID-19 will not be fully understood for some time, this health crisis has the potential to further impact and accelerate the existing physical inactivity pandemic (Hall et al., 2020). For this reason, ACSM recently published PA guidelines emphasizing the importance of staying active while taking precautions to ensure safety in the midst of the COVID-19 pandemic (Denay et al., 2020).

Physicians represent a key figure in the dissemination of PA recommendations to a broad segment of the population, with evidence that those who are guided to perform exercise by their physician, perform more moderate-to-vigorous intensity PA than those who do not receive any exercise prescription (Taylor, 2014). This can be particularly important for older individuals who are less likely to engage in PA, compared to their younger counterparts (Gavin et al., 2014). Where an individual has previously acquired knowledge regarding safe, functional, and simple PA, medical clearance or a health appraisal prior to engaging in novel forms of PA might not be necessary. It is important to note that not all age groups will show the same level of eagerness to engage in a new form of PA (Gavin et al., 2014). Instead, varying the intensity may provide the stimulus and novelty required to increase PA adherence and elicit significant health benefits. One plausible approach might be interval training, whereby the intensity of movement is intermittently varied. Studies have shown that interval training can improve enjoyment (Stork et al., 2017), affective response (Niven et al., in press), and mental and physiological adaptations (Martland et al., 2020) in comparison to continuous moderate intensity exercise. Finally, enjoyment is an important factor mediating the level of PA adherence (Wankel, 1993; Jekauc, 2015).

Knowing that non-communicable diseases are the leading threat to public health on a global scale (Thornton et al., 2016), the importance of regular PA, as a therapeutic agent tackling non-communicable diseases, cannot be overestimated. To increase the overall population rate of adherence to ACSM/AHA guidelines, we believe that novelty can play a key part in helping individuals reach these recommendations. Accordingly, physicians, researchers, and practitioners should place a special emphasis on novelty, as, potentially, one of the key determinants contributing to PA adherence. Ultimately, engaging in PA that is enjoyable, improves health and wellbeing, and provides continual novel stimulus, will lead to improved engagement and adherence over time.

## Author Contributions

NL, AG, AB, and PD contributed to conception and design of the study and wrote the first draft of the manuscript. SM, SC, KP, and RR wrote sections of the manuscript. All authors contributed to manuscript revision, read, and approved the submitted version.

## Conflict of Interest

The authors declare that the research was conducted in the absence of any commercial or financial relationships that could be construed as a potential conflict of interest.
